# Effects of risk perception and agricultural socialized services on farmers' organic fertilizer application behavior: Evidence from Shandong Province, China

**DOI:** 10.3389/fpubh.2023.1056678

**Published:** 2023-01-23

**Authors:** Zhong Ren

**Affiliations:** Business School, Shandong Normal University, Jinan, China

**Keywords:** organic fertilizer application behavior, risk perception, agricultural socialized services, moderating effect, technical risk, market risk

## Abstract

**Introduction:**

The application of organic fertilizer is an important measure to control agricultural non-point source pollution, improve the quality of cultivated land and enhance the degree of agricultural green development.

**Methods:**

Based on the survey data of sample farmers in Shandong Province, China, the binary Probit model is used to analyze the influence of risk perception and agricultural socialized services and their interaction on farmers' organic fertilizer application behavior, and further analyze the difference of influence between groups of risk perception and agricultural socialized services on farmers' organic fertilizer application behavior with different characteristics.

**Results and discussion:**

We found that risk perception has a significant negative impact on farmers' organic fertilizer application behavior. Farmers with stronger technical risk perception and market risk perception are less likely to apply organic fertilizer. Agricultural socialized services have a significant positive impact on farmers' organic fertilizer application behavior, and can effectively alleviate the inhibitory effect of risk perception on farmers' organic fertilizer application behavior. The roles of risk perception and agricultural socialized services vary greatly among different farmer groups. For older generation, large-scale and pure agricultural farmers, agricultural socialized services can more effectively alleviate the inhibitory effect of risk perception on organic fertilizer application behavior.

## 1. Introduction

Fertilizer is an important input factor in agricultural production, and has made great contributions to improving agricultural production efficiency, ensuring agricultural product supply, and maintaining food security (1, 2). However, relevant research shows that the long-term excessive and inefficient application of chemical fertilizer not only leads to the deterioration of the ecological environment in terms of soil compaction, eutrophication of water bodies, and greenhouse gas emissions, but also poses hidden dangers to the quality and safety of agricultural products and human health (3–5). China is the country with the largest population in the world. To ensure food security, China's annual use of chemical fertilizers ranks first in the world, accounting for one third of the world. In 2021, the application amount of chemical fertilizer per hectare of crops in China reached 362.41 kg, far higher than the world average (120 kg). The influence of excessive use of chemical fertilizer on land in China has been very prominent (6, 7). According to the Bulletin on the Quality Grade of Cultivated Land in China, the pollution area of cultivated land accounts for 19.4%, the cultivated land area of middle and low yield fields accounts for 68.76% of the total cultivated land area. For this reason, China government has successively issued a series of policies to guide the reduction of chemical fertilizers. In 2015, the Ministry of Agriculture released the “Action Plan for Zero Growth of Fertilizer Use by 2020.” The Central No. 1 document from 2016 to 2020 has emphasized the need to carry out the work of reducing and replacing chemical fertilizer for five consecutive years to enhance the sustainable development of agriculture. It can be seen that how to effectively promote the reduction of chemical fertilizer is a major practical problem facing the development of China's agricultural modernization.

As an environmentally friendly agricultural production material, organic fertilizer has excellent characteristics such as complete nutrients, stable fertilizer efficiency, improvement of soil structure, and protection of ecological environment. It plays an important role in the quality and safety of agricultural products and the reduction of chemical fertilizer (8, 9). Although the Chinese government has been promoting the technology of replacing chemical fertilizer with organic fertilizer in recent years, the actual application rate of organic fertilizer is not satisfactory, and the utilization rate is only 40.2% (10). Organic fertilizer application behavior (OFAB) refers to the investment decision-making behavior of agricultural production and management entities in applying organic fertilizers such as farmyard manure and commercial organic fertilizer in agricultural production in order to maximize profits (11, 12). As the main body of agricultural production and operation, farmers have the direct decision-making power of fertilizer application. It is of great significance to clarify their OFAB and key influencing factors for the effective promotion of organic fertilizer.

Overall, the factors affecting farmers' OFAB are diverse and complex. In terms of individual characteristics, gender, age, and educational level have an impact on OFAB (13, 14). In terms of family endowment characteristics, land size, income level, and family size all affect OFAB (15, 16). In terms of cognitive characteristics, there is a positive correlation between green cognition, technical cognition and OFAB (17, 18). In terms of the external environment, policy subsidies, technical training, publicity and education, and social capital have a significant positive impact on farmers' OFAB (19, 20). In addition to the above factors, affected by factors such as the increase in global extreme weather and food price fluctuations, the current decision-making of farmers' production behavior is faced with multiple uncertain risks, and farmers' subjective judgments on various risk factors constitute their risk perception (21). Studies have confirmed that risk perception, as an important research tool for evaluation, decision-making and behavior, has an important impact on farmers' pesticide use behavior and biosafety behavior (22, 23), but few people pay attention to the role of risk perception in the decision-making of OFAB.

In addition, in recent years, China's agricultural socialized services have developed rapidly and are playing an increasingly important role in agricultural production. Relying on its professional and technical personnel, green production materials, low cost and market competitive advantages, agricultural socialized services can alleviate the problems of high risk, high cost and insufficient technical management ability faced by individual farmers in technology adoption, and can significantly promote farmers' green production technology adoption (24, 25). For example, land trusteeship services have promoted farmers to adopt soil testing and formula fertilization techniques (26), and specialized plant protection services have significantly reduced the intensity of pesticide application (27). Then, under the background of the continuous improvement of the agricultural socialized services, can agricultural socialized services have an impact on farmers' OFAB? What role does it play in the impact of risk perception on farmers' OFAB? These questions have yet to be confirmed and answered.

The contributions of this study are: first, we construct a theoretical analysis framework for farmers' OFAB from the perspective of risk perception at the internal level and agricultural socialized services at the external level, rather than just focusing on the impact of risk perception or agricultural socialized services, which provides a new research perspective for the study of OFAB. Second, based on field survey data, the relationship between risk perception, agricultural socialized services and OFAB was empirically verified. This provides theoretical support and practical guidance for the promotion of organic fertilizer application.

The structure of this paper is arranged as follows: the first part is the proposal of the problem; the second part builds a theoretical framework, explains the micro-mechanism of risk perception and agricultural socialized services affecting farmers' OFAB, and puts forward research hypotheses; the third part introduces the measurement model setting and data sources, and perform descriptive statistical analysis on the data; the fourth part reports and analyzes the estimated results; the fifth part is the research conclusions and policy implications.

## 2. Theoretical basis

### 2.1. Perceived risk and OFAB

Risk perception refers to the behavior subject's feeling and cognition of various uncertain consequences that are or may affect them, emphasizing the influence of behavior subject's feelings and intuitive judgment on their cognition (28). When the behavior subject is in a state of risk, it will affect their psychology, and usually adopt risk-averse behaviors to relieve inner anxiety and pressure (29). Therefore, risk perception plays an important role in individual decision-making and can be used as an explanatory variable for decision-making.

Based on the goal of maximizing profits, rational farmers will consider and judge the expected benefits when faced with the choice of whether to apply organic fertilizer, while farmers' understanding of benefits is largely reflected in the increase of output and price (6, 30), accompanied by the technical risk and market risk of OFAB (31). Specifically: firstly, technical risk. Since organic fertilizer itself has the characteristics of low application efficiency, high input cost, long benefit period and large uncertainty, reducing the application of chemical fertilizer may have the risk of reducing production (32, 33). The second is market risk. As China's green agricultural product market system has not yet been perfected, the quality information in the agricultural product market is asymmetric, so that farmers who produce organic agricultural products cannot obtain the benefits of “quality premium” (34, 35). Therefore, as a passive receiver of market price, small farmers have certain market risk when applying organic fertilizer. Although the above risks exist objectively, compared with the actual occurrence of risks, farmers' production behavior decisions are more easily influenced by subjective risk perception (36). Different farmers have different endowments, which will lead to differences in their perception and evaluation of organic fertilizer application, and then affect their OFAB. Under the goal of risk minimization, the higher the farmers' perceived risk, the lower the possibility of applying organic fertilizer. Accordingly, this paper puts forward hypothesis H_1_:

H_1_: Risk perception significantly affects farmers' OFAB.

### 2.2. Agricultural socialized services and OFAB

In a broad sense, agricultural socialized services refer to the support services provided by social and economic organizations or individuals for pre-production, production, and post-production in agriculture (37). In China, the main bodies of agricultural socialized services include leading enterprises, agricultural cooperatives, and village collectives. Most studies think that agricultural socialized services can effectively replace family labor force, and has positive effects on increasing rice yield per unit area (38), increasing family income (39), reducing production cost (40), improving farmers' welfare and increasing production technical efficiency (41, 42). More specifically, agricultural socialized services have an important influence on farmers' decision-making of production behavior.

As far as the application of organic fertilizer is concerned, the agricultural socialized services are embodied in: firstly, to achieve high-quality and low-price agricultural supplies before production. In the agricultural market, there are many kinds of organic fertilizers, and there are differences among them in usage, content and proportion of effective ingredients, and absorption and utilization efficiency of crops. It is difficult for farmers to purchase high-quality organic fertilizer through experience accumulation (43). Agricultural socialized services have the function of unified agricultural material procurement service, which can ensure fertilizer quality on the one hand, and reduce the cost for members to obtain information on fertilizer quality on the other hand, thereby promoting farmers' application (44). Secondly, technical guidance or training is provided during production. The application of organic fertilizer is a knowledge-intensive technology and requires a high level of knowledge of farmers. However, in the state of decentralized operation in China, a single farmer cannot master the professional knowledge of organic fertilizer application based on his own experience (45). Agricultural socialized services can provide farmers with high-quality and sufficient agricultural technical guidance through various forms such as technical training, printing and distributing technical materials, and holding professional conferences to improve the utilization level of organic fertilizer. Finally, provide high-quality and preferential sales services. Compared with small farmers, the main bodies of agricultural socialized services have absolute advantages in expanding sales channels and enhancing the market premium capacity of agricultural products, and can transfer part of the risk of uncertain net profit faced by farmers to themselves (46). Farmers are willing to increase the application of organic fertilizers out of the pursuit of high-quality and high-price benefits and the avoidance of sales risks. Accordingly, this paper puts forward hypothesis H_2_:

H_2_: Agricultural socialized services significantly affect farmers' OFAB.

### 2.3. The moderating role of agricultural socialized services

Attitude-situation-behavior theory shows that the influence of individual attitude on behavior will be influenced by situational factors (47). Risk perception, as a subjective feeling, cognition and judgment of the behavior subject, is formed based on the individual's objective experience and situation. Individuals in different situations have very different risk perceptions (48). As mentioned above, farmers may face technical risk and market risk when applying organic fertilizer. The higher the risk level perceived by farmers, the less likely they are to apply organic fertilizer. Agricultural socialized services can help reduce farmers' risk perception. In terms of technical risk, agricultural socialized services ensure the quality of organic fertilizer through unified procurement, and technical guidance in production can accurately grasp the technical characteristics of organic fertilizer application, thus alleviating the problems of high risk, high cost and insufficient technical management ability faced by individual farmers in technology adoption (24). In terms of market risk, farmers can't accurately predict consumers' requirements for agricultural products during production, while agricultural socialized services are consumer-oriented, which has the motivation to make technical improvements in the production process, and can obtain high returns by transmitting quality and safety information to consumers, thus reducing the market risk of farmers applying organic fertilizer (49). Accordingly, this paper proposes hypothesis H_3_:

H_3_: Agricultural socialized services have a moderating role in the impact of risk perception on farmers' OFAB.

**Figure 1:**
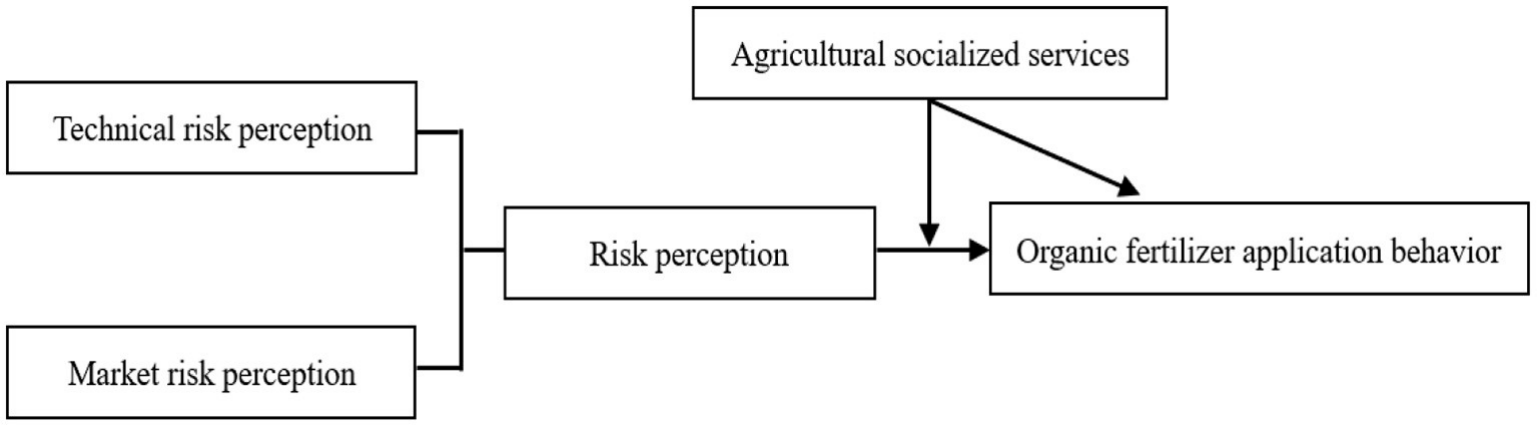
Theoretical model.

Based on the above research hypothesis, the theoretical model is shown in Figure 1.

## 3. Study design

### 3.1. Data collection and analysis

The data used in this paper comes from our investigation in Shandong Province, China from September to November, 2021. On the one hand, the choice of investigation area is based on Shandong Province as the largest vegetable province in China, and its vegetable output accounts for about 11.3% of the whole country. On the other hand, due to the large amount of chemical fertilizer application in Shandong Province, the phenomenon of environmental pollution caused by excessive application of chemical fertilizer is more prominent.

The minimum recommended size of the survey sample was calculated as 324 people with a confidence level of 95% and a margin of error of 5% (50). Before the formal investigation, a small-scale preliminary investigation was conducted in Jinan City, and 10 vegetable growers were randomly selected to conduct questionnaire interviews, and the questionnaires were revised and improved. In order to fully reflect the principle of random sampling and the balance of sample distribution, in the selection of formal research sites, first of all, in combination with the delineation of key vegetable production counties in Shandong Province, and at the same time fully consider the differences in topography and landforms, and pay attention to the balance of the selection of samples with different terrains. Finally, six sample counties including Shouguang, Xinxian, Zhangqiu, Feicheng, Lanling, and Pingdu were selected. Then, 2 townships (streets) were randomly selected from each sample county (city, district), then 2 villages were randomly selected from the sample townships (streets), and finally at least 20 farmers were randomly selected from each village for questionnaire survey.

Interviews are mainly conducted in farmers' homes or village committee offices. Before the interview, we first told the farmers that the interview was completely anonymous, the survey results were only used for academic research, and each interviewee signed an informed consent form. Then, the researcher asked the farmers about the application of organic fertilizer, risk perception and agricultural socialized services. Farmers who have no obstacles to fill out the questionnaire will fill it out by themselves. For those who can't read and have difficulty in understanding, investigators will help them fill it out. The interview time of each farmer ranges from 15 to half an hour. In addition, we also sought the help of the local agricultural department and conducted a questionnaire survey on farmers who were inconvenient to contact. After the interview, we sorted out the questionnaires and deleted the invalid ones. A total of 500 questionnaires were completed, 494 were recovered, and 480 valid questionnaires were recovered, with an effective recovery rate of 97.17%.

The survey results (Table 1) show that the farmers interviewed are mainly men, accounting for 88.13%. Most of them are between 51 and 60 years old, accounting for 54.58% of the total sample. The average length of education is 6.80 years, mainly from junior high school and below. The sample households with two labors accounted for 76.67%. The average cultivated land area per household is 7.1 mu, with few large planters. The annual household income is mostly 50,000–100,000 yuan, accounting for 39.37%. Among the farmers interviewed, 302 households used organic fertilizer, accounting for 62.92% of the total sample. These data are basically consistent with the results of the third agricultural census in China, indicating that the survey data of this study is representative.

**Table 1:** Basic information of sample farmers.

**Variable**	**Category**	**Frequency**	**Proportion**	**Variable**	**Category**	**Frequency**	**Proportion**
Gender	Male	423	88.13	Number of labor force	1	32	6.67
	Female	57	11.87		2	368	76.67
Age	≤ 40	23	4.79	Annual income/million RMB	≥3	80	16.66
	41–50	114	23.75		≤ 5	141	29.38
	51–60	262	54.58		5–10	189	39.37
	≥60	83	17.28		≥10	150	31.25
Education level	Primary school	128	26.67	Planting scale/mu	≤ 5	142	29.58
	Junior high school	158	32.92		6–10	192	40.00
	Senior high school	86	17.91		11–20	81	16.88
	Junior college and above	108	22.50		≥21	65	13.54

### 3.2. Model setting

The dependent variable “OFAB” in this study includes two choices: “application” and “no application”, which can be represented by response 1 and response 0, respectively. When there are multiple independent variables in the binary model, the model can be defined in matrix form:


(1) $$\begin{array}{l} y_{i}=\beta X_{i}+\varepsilon_{i} \end{array}$$

In the formula (1), *y*_*i*_ is the decision-making explained variable with observed values of 1 and 0; *X*_*i*_ is the variable to be explained, including the attribute of the selected object data and the attribute of the selected subject; *β* is the parameter to be estimated; *ε*_*i*_ is a random interference term.

The probability distribution of random disturbance *ε*_*i*_ determines the specific form of binary choice model: Probit model and Logit model. Probit model pays more attention to standard normal distribution, while Logit model pays more attention to logical distribution. Because normal distribution is considered as the natural and first choice of any distribution, Probit model is the most widely used binary choice model. Therefore, a binary Probit model is constructed with the following expression:


(2) $$\begin{array}{l} y_{i}=\alpha_{i}+\beta_{i1}RP+\beta_{i2}ASS+\beta_{i3}RP\times ASS+\beta_{i4}Control_{ij}+\varepsilon_{i} \end{array}$$

In the formula (2), *y*_*i*_ is whether or not the farmer applies organic fertilizer. The value that has been applied is “1,” and the value that has not been applied is “0.” *RP* is risk perception, *ASS* is agricultural socialized services, *RP* × *ASS* is the interaction term between risk perception and agricultural socialized services, *Control*_*ij*_ is the *j* control variable of the *i* farmer. *β* is the coefficient to be estimated, *β*_*i*1_, *β*_*i*2_, *β*_*i*4_ are used to judge the impact of risk perception, agricultural socialized services and control variables on farmers' OFAB, respectively, *β*_*i*3_ is used to judge the moderating role of agricultural socialized services in the impact of risk perception on OFAB. α_*i*_ is a constant term. *ε* is a random error term.

### 3.3. Variable selection

(1) Explained variables. The explanatory variable in this paper is the farmers' OFAB, when the farmers apply organic fertilizer, the value is 1; otherwise, the value is 0.
(2) Core explanatory variables. There are two core variables in this paper, namely risk perception and agricultural socialized services. For risk perception, it is measured from two dimensions: technical risk and market risk. Referring to relevant studies (51, 52), two items were set up, “the effect of applying organic fertilizers on yield reduction” and “the agricultural products applied with organic fertilizers cannot be sold at high prices” to represent technical risk and market risk, and use a five-level Likert scale to measure. For agricultural socialized services, referring to relevant research (53), it is analyzed from the design of three production links: pre-production, mid-production and post-production. Questions in the questionnaire design include “Whether there are organizations or individuals that provide unified purchase of organic fertilizer services,” “Whether organizations or individuals provide organic fertilizer technical guidance services,” “Whether there are services for unified sales of organic agricultural products,” the options are all “Yes” or “No.” If the farmer participates in at least one agricultural socialized service, the value is 1, and if the farmer does not participate in any agricultural socialized service, the value is 0.
(3) Control variables. Referring to relevant studies (54–56), gender, age, education level, health status, and concurrent employment were selected to reflect individual characteristics of farmers; total household income, number of household agricultural laborers, planting scale, and soil fertility were selected to reflect family management characteristics; whether the application of organic fertilizer is subsidized, whether the government has carried out publicity on organic fertilizer, whether the government has set up demonstration households of organic fertilizer application technology to reflect the characteristics of the policy environment. The specific characterization and related description of each variable are shown in Table 2.

**Table 2:** Variable definition and descriptive statistics.

**Variable name**	**Variable definition and assignment**	**Mean**	**Standard deviation**
OFAB	Whether to apply organic fertilizer: yes = 1; no = 0	0.63	0.37
Technical risk	Effect of applying organic fertilizer on yield reduction: no impact = 1; less impact = 2; general = 3; larger impact = 4; very big impact = 5	3.15	0.89
Market risk	Agricultural products applying organic fertilizer cannot be sold at a high price: totally disagree = 1; disagree = 2; general = 3; agree = 4; strongly agree = 5	3.56	1.04
Agricultural socialized services	Participate in at least one agricultural socialized service: yes = 1; no = 0	0.52	0.48
Gender	Female = 0; male = 1	0.88	0.32
Age	Actual age of interviewee (years)	54.12	9.67
Educational level	No school = 1; primary school = 2; junior high school = 3; high school or junior college = 4; undergraduate and above = 5	3.10	0.77
Health status	Very poor = 1; poor = 2; general = 3; good = 4; very good = 5	3.63	0.86
Concurrent employment	Whether to concurrent employment: yes = 1; no = 0	0.77	0.44
Total household income	Actual household income in 2021 (10,000 yuan)	5.63	3.21
Number of household agricultural laborers	Number of laborers engaged in agricultural production (person)	2.22	0.58
Planting scale	Planting area in 2021 (mu)	7.11	9.45
Soil fertility	Poor = 1; general = 2; good = 3	2.16	0.62
Subsidy policy	Whether the government subsidizes the application of organic fertilizer: yes = 1; no = 0	0.34	0.68
Publicity policy	Whether the government has publicized the organic fertilizer technology: yes = 1; no = 0	0.48	0.85
Demonstration policy	Whether the government has set up demonstration households of organic fertilizer application technology: yes = 1; no = 0	0.24	0.27

## 4. Empirical results and discussion

### 4.1. Multicollinearity test

Considering the possible internal correlation between variables, in order to ensure the validity and rationality of the study, a multicollinearity test was performed on each explanatory variable before the formal regression analysis. The results show that the largest variance inflation factor is 1.73, which is far <10, indicating that the degree of collinearity among the explanatory variables is in a reasonable range and satisfies the principle of independence.

### 4.2. Reliability and validity test

Reliability refers to the consistency of data test results. Cronbach's α reliability coefficient and CR value are the frequently used analysis methods at present, which are statistically analyzed by stata16.0 software, as shown in Table 3. Cronbach's α values of each dimension are all above 0.8, ranging from 0.923 to 0.961, respectively, and the combined reliability is all above 0.8, ranging from 0.902 to 0.937, respectively, indicating good reliability.

**Table 3:** Reliability and validity test results.

**Variable**	**Cronbach's α**	**CR**	**KMO**	**Bartlett**
Risk perception	0.923	0.911	0.917	0.000
Agricultural socialized services	0.940	0.902	0.912	0.000
OFAB	0.961	0.937	0.904	0.000

The purpose of validity test is to measure the accuracy of measurement. KMO and Bartlett were used for validity test. Experience shows that KMO > 0.9 indicates that data analysis can be carried out to a great extent, 0.9 > KMO > 0.8 indicates that it is suitable, 0.8 > KMO > 0.7 is acceptable, and below 0.5, it is necessary to analyze the questionnaire structure or consider the theory and then revise it again. The results show that the overall KMO test value is 0.915, which is higher than the average fitness value of 0.7, and the significance of Bartlett statistical value is 0.000 < 0.001, which indicates that the scale has good validity.

### 4.3. Analysis of benchmark regression results

Table 4 lists the estimated results of the impact of risk perception and agricultural socialized services on farmers' OFAB. Among them, Model 1 is the estimation result that only includes control variables, and Model 2 is the estimation result that includes risk perception and agricultural socialized services. From the regression results, the Pseudo *R*^2^ of Model 2 increases to 0.349, which has stronger explanatory power. Therefore, this study mainly discusses the estimation results of Model 2.

**Table 4:** Benchmark regression results.

**Variable**	**Model 1**		**Model 2**	

	**Coefficient**	**Standard error**	**Coefficient**	**Standard error**
Technical risk	–	–	−0.767^***^	0.294
Market risk	–	–	−0.824^***^	0.276
Agricultural socialized services	–	–	0.443^***^	0.242
Gender	0.165	0.074	0.143	0.079
Age	0.063	0.013	0.048	0.018
Educational level	0.737^**^	0.217	0.482^**^	0.228
Health status	0.009	0.005	0.008	0.005
Concurrent employment	−0.037	0.131	−0.032	0.138
Total household income	0.306^***^	0.317	0.367^**^	0.351
Number of household agricultural laborers	0.105	0.198	0.116	0.212
Planting scale	0.429	0.228	0.370	0.248
Soil fertility	−0.204^*^	0.329	−0.426^*^	0.255
Subsidy policy	0.528	0.407	0.494	0.452
Publicity policy	0.676^*^	0.349	0.665^**^	0.392
Demonstration policy	0.280	0.117	0.256	0.127
Prob > chi^2^	0.000		0.000	
Pseudo *R*^2^	0.286		0.349	

* ,

** ,

*** Indicate significant at the 10, 5, and 1% levels, respectively.

(1) Risk perception. The technical risk has a significant negative impact on farmers' OFAB at the 1% level, indicating that the higher the farmers' perception of the technical risk of organic fertilizer, the lower the possibility of applying organic fertilizer. Because organic fertilizer has the characteristics of slow fertilizer efficiency, and the application of organic fertilizer requires a certain knowledge reserve, there is a potential risk that farmers with generally low knowledge level will reduce their output due to improper technology adoption. When farmers perceive the technical risk of organic fertilizer application, they tend to reduce the possibility of applying organic fertilizer out of risk aversion. Market risk has a significant negative impact on farmers' OFAB at the 1% level, indicating that the higher farmers' perception of market risk of organic fertilizer, the lower the possibility of applying organic fertilizer. Under the realistic background that the market system of organic agricultural products is not perfect, organic agricultural products can't get the premium of quality. At the same time, the application of organic fertilizer requires farmers to invest more manpower and material capital, which makes farmers' perception of the market risk of organic fertilizer stronger, thus causing resistance to the application of organic fertilizer.
(2) Agricultural socialized services. Agricultural socialized services have a significant positive impact on farmers' OFAB at the level of 1%, indicating that the more agricultural socialized services farmers get, the higher the possibility of applying organic fertilizer. Compared with a single small farmer, agricultural socialized service organizations have a higher ability to apply organic fertilizer. The more farmers participate in agricultural socialized services, it is equivalent to being given a higher ability to apply organic fertilizer in a short time, which makes the investment in applying organic fertilizer more sufficient and efficient, and reduces the transaction cost of application. At the same time, agricultural socialized services can also improve the output efficiency of farmers after applying organic fertilizer through targeted marketing and other upstream and downstream links and product premium capacity.
(3) Control variables. Education level at 5% level has a significant positive impact on farmers' OFAB. Education determines the knowledge structure of farmers, and indirectly affects their ability to obtain technical support, means of production and other elements. Therefore, the higher the education level of farmers, the more favorable it is to apply organic fertilizer. Soil fertility at 10% level has a significant negative impact on farmers' OFAB. Because organic fertilizer has a long effect and can improve the physical and chemical properties of soil, but at the same time it has the disadvantages of large application amount, slow effect and high demand for labor force. When farmers think that the fertility of their cultivated land is good, the possibility of applying organic fertilizer is lower for the sake of saving labor cost and economic cost. The total household income at the level of 5% has a significant positive impact on farmers' OFAB. The families with more total income can bear the economic cost of organic fertilizer and have certain anti-risk ability, thus increasing the possibility of farmers applying organic fertilizer. Publicity policy has a significant positive impact on farmers' OFAB at 5% level. Through free explanation and publicity, the government can make farmers realize the harm of excessive application of chemical fertilizer, improve farmers' understanding of the application technology and advantages of organic fertilizer, and thus promote the application of organic fertilizer.

### 4.4. Moderating effect analysis

In order to further study the moderating effect of agricultural socialized services between farmers' risk perception and OFAB, the interaction between risk perception and agricultural socialized services was incorporated into the model, and the estimated results are shown in Table 5. The interaction item between technology risk and agricultural socialized services, and the interaction item between market risk and agricultural socialized services have a significant positive impact on farmers' OFAB at the level of 1 and 5%, respectively. Because the risk perception coefficient is negative and its interaction is positive, it shows that agricultural socialized service can alleviate the inhibition of risk perception on OFAB. On the one hand, the more farmers participate in agricultural socialized services, the more they can realize the optimal decision of resource allocation, thus alleviating the factor endowment constraint of their behavior of applying organic fertilizer. On the other hand, the more agricultural socialized services farmers participate in, the more favorable it is to reduce the transaction costs caused by the contradiction between small production and big market, thus realizing the safety and reliability of agricultural materials market and the symmetry of agricultural products market information.

**Table 5:** Test results of the moderating effect of agricultural socialized services.

**Variable**	**Coefficient**	**Standard error**
Technical risk × Agricultural socialized services	0.604^***^	0.267
Market risk × Agricultural socialized services	0.526^**^	0.184
Control variables	Controlled	
Prob > chi^2^	0.000	
Pseudo *R*^2^	0.326	

** ,

*** Indicate significant at the 5, 1% levels, respectively.

### 4.5. Heterogeneity analysis

While exploring the effects of risk perception and agricultural socialized services on OFAB, we also care about the roles of these two core variables among different groups. To this end, this paper subdivides and empirically tests the sample farmers by age, planting scale, and concurrent employment. Referring to related research (57), farmers were divided into older generation group (born before 1975) and new generation group (born in 1975 and later). According to the World Bank's definition standard of small farmers with an area of <2 hm2 of arable land per household, farmers are divided into small-scale group and large-scale group. Based on farmers' concurrent employment situation, farmers are divided into concurrent employment group and pure agricultural group. The specific results of the regression are shown in Table 6.

**Table 6:** Regression results of different groups of farmers.

**Variable**	**Age**		**Planting scale**		**Concurrent employment**	

	**New generation**	**Old generation**	**Small-scale**	**Large-scale**	**Concurrent employment**	**Pure agricultural**
Technical risk	−0.214	−1.320^***^	−0.978^***^	−0.626^*^	−0.448	−1.193^***^
Market risk	−0.333	−0.949^***^	−0.770^**^	−1.004^***^	−0.505	−0.984^**^
Agricultural socialized services	0.299^**^	0.618^***^	0.419^***^	0.477^**^	0.196^**^	0.885^***^
Technical risk × Agricultural socialized services	0.376^*^	1.003^**^	0.518^*^	0.823^**^	0.572	0.693^*^
Market risk × Agricultural socialized services	0.310	0.626^**^	0.422^*^	0.754^**^	0.451	0.637^**^
Control variables	Controlled	Controlled	Controlled	Controlled	Controlled	Controlled
Pseudo *R*^2^	0.261	0.306	0.295	0.275	0.311	0.280

* ,

** ,

*** Indicate significant at the 10, 5, and 1% levels, respectively.

(1) Different age groups. Both technical risk and market risk have a significant negative impact on the older generation of farmers, but neither has a significant impact on the new generation of farmers. This is because the old generation farmers are more conservative in their decision-making and have more concerns about whether the OFAB will bring losses, while the new generation farmers have more channels to obtain information and are more likely to accept new things. Agricultural socialized services have a greater impact on the older generation of farmers, and play a stronger role in alleviating the technical and market risks of the older generation of farmers. Due to the dual constraints of “physical energy effect” and “knowledge effect,” the older generation of farmers is more inclined to make up for the shortage of labor input and knowledge shortage by means of agricultural socialized services, so they are more dependent on agricultural socialized services.
(2) Different planting scale groups. Market risk has a more significant impact on large-scale farmers, and technical risk has a more significant impact on small-scale farmers. Agricultural socialized services have a greater impact on large-scale farmers, and it play a stronger role in alleviating the technical risk and market risk of large-scale farmers. The social network structure of large-scale farmers is more complex, and there are more network members, so the quantity and quality of information they get are higher. Therefore, it is easier to understand and obtain the services of agricultural socialized organizations, and reduce adverse selection and moral hazard caused by information asymmetry.
(3) Different concurrent employment groups. Both technical risk and market risk have a significant negative impact on pure agricultural farmers, but no significant impact on concurrent employment farmers. Agricultural socialized services have a greater impact on pure agricultural farmers, and play a stronger role in alleviating the technical risk and market risk of pure agricultural farmers. A large proportion of the income of concurrent employment farmers comes from non-agricultural activities, and they pay less attention to the productivity and sustainable utilization of cultivated land, and are less willing to spend time and energy on the OFAB. Pure agricultural farmers are more dependent on land, more sensitive to the risk of applying organic fertilizer, and more inclined to use the specialized services of agricultural socialized service organizations to mitigate risks.

### 4.6. Robustness test

In order to further test the reliability of the above model results, this paper introduces risk perception, agricultural socialized services and their interaction terms into the Logit model for regression. The results are shown in Table 7. It can be seen that technical risk and market risk have a significant negative impact on OFAB at the level of 1%. Agricultural socialized services have a significant positive impact on farmers' OFAB at the 1% level. The interaction item between technology risk and agricultural socialized services, and the interaction item between market risk and agricultural socialized services have a significant positive impact on farmers' OFAB at the level of 1 and 5%, respectively. The estimation results are consistent with the regression results of the Probit model in significance and direction of action, indicating that the conclusions of this study are relatively robust.

**Table 7:** Robustness test estimation results.

**Variable**	**Coefficient**	**Standard error**
Technical risk	−0.779^***^	0.296
Market risk	−0.821^***^	0.276
Agricultural socialized services	0.445^***^	0.242
Technical risk × Agricultural socialized services	0.611^***^	0.270
Market risk × Agricultural socialized services	0.521^**^	0.178
Control variables	Controlled	Controlled
Pseudo *R*^2^	0.352	

^**^, ^***^Indicate significant at the 5 and 1% levels, respectively.

## 5. Conclusions and policy enlightenment

How to popularize the application of organic fertilizer plays an important role in promoting the green and sustainable development of agriculture. Based on the survey data of Shandong Province, China, this study uses Probit regression model to explore the influence of risk perception and agricultural socialized services on farmers' OFAB. The empirical results show that both technical risk perception (0.767) and market risk perception (0.824) have significant negative effects on OFAB. When farmers' perception of technical risk and market risk is lower, they are more willing to apply organic fertilizer. On the other hand, agricultural socialized services (0.443) have a significant positive impact on OFAB. The more agricultural socialized services farmers get, the higher the possibility of applying organic fertilizer. In addition, among the influencing paths of technical risk perception and market risk perception to OFAB, agricultural socialized services have played an adjusting role of 60.4 and 52.6% respectively, which indicates that agricultural socialized services can effectively alleviate the inhibition of risk perception to farmers' OFAB. This study also found that the impact of risk perception and agricultural socialized services on farmers' OFAB is significantly heterogeneous. Specifically, older generation, large-scale and pure farmers are more susceptible to risk perception and agricultural socialized services. Generally speaking, this study provides a comprehensive assessment of the impact of risk perception and agricultural socialized services on OFAB. These results are of great significance to understand how risk perception and agricultural socialized services affect OFAB, which is helpful to enrich the research on OFAB.

Although this research is based on China, some developing countries in South Africa and South Asia are also facing the problems of soil pollution and environmental damage caused by excessive use of chemical fertilizers (58, 59). With the development of information technology, farmers' geographical restrictions are becoming more and more diluted (20), while the green production technology has no international boundaries (60, 61). Therefore, the analytical framework and conclusion of this study also have important practical enlightenment for other developing countries and even developed countries to promote OFAB: (1) Improve the market price formation mechanism of agricultural products and improve the quality certification system of agricultural products. Through the brand building of green agricultural products, a green credit system for agricultural products will be constructed, and a market environment of “high quality and good price” for agricultural products will be formed, so as to solve the “lemon effect” caused by information asymmetry in the current agricultural product market, thereby reducing the market risk of farmers' OFAB. (2) Strengthen the innovation and research and development of organic fertilizer technology, and focus on solving the technical defects of organic fertilizer. At the same time, the publicity and training of organic fertilizer technology and field guidance should be strengthened, and the interpretation of relevant information such as application methods, operation processes and application effects of organic fertilizer should be done well, so as to enhance farmers' objective cognition and operational proficiency of organic fertilizer technology, and thus reduce the technical risk of farmers' OFAB. (3) Vigorously promote the development of agricultural socialized services, expand service contents and service targets, and constantly improve service quality. Give full play to material supply, technical guidance, and agricultural product sales services to resolve risk perception of farmers' OFAB. At the same time, we need to pay attention to the “matching effect” of agricultural socialized services and different groups of farmers. (4) Different groups have different goals in pursuing agricultural management, and their focus in the process of applying organic fertilizer is also different. Therefore, before the process of technical publicity, training and promotion, we can try to classify farmers, select different service contents according to the characteristics of different groups of farmers, and carry out differentiated incentives.

There are also some shortcomings in this study: firstly, the data analysis in this study is based on non-experimental cross-sectional data, which can't analyze the dynamic changes of farmers' OFAB. In the follow-up research, we can consider establishing long-term tracking panel data, so as to improve the reliability of the measurement results. Secondly, farmers' OFAB is influenced by many factors. This study only tries to discuss two factors: farmers' risk perception and agricultural socialized services, and the choice of explanatory variables may not be complete. If future research can incorporate more factors into the theoretical model, a more comprehensive conclusion may be drawn.

## Data availability statement

The raw data supporting the conclusions of this article will be made available by the authors, without undue reservation.

## Ethics statement

The studies involving human participants were reviewed and approved by Shandong Normal University. The patients/participants provided their written informed consent to participate in this study.

## Author contributions

The author confirms being the sole contributor of this work and has approved it for publication.
